# Blood glucose may be an alternative to cholesterol in CVD risk prediction charts

**DOI:** 10.1186/1475-2840-12-24

**Published:** 2013-01-25

**Authors:** Julia Braun, Matthias Bopp, David Faeh

**Affiliations:** 1 Institute of Social and Preventive Medicine (ISPM), University of Zurich, Hirschengraben 84, 8001, Zurich, Switzerland

**Keywords:** Cardiovascular disease mortality, Risk score, Risk prediction, Blood cholesterol, Blood glucose

## Abstract

**Background:**

Established risk models for the prediction of cardiovascular disease (CVD) include blood pressure, smoking and cholesterol parameters. The use of total cholesterol for CVD risk prediction has been questioned, particularly for primary prevention. We evaluated whether glucose could be used instead of total cholesterol for prediction of fatal CVD using data with long follow-up.

**Methods:**

We followed-up 6,095 men and women aged ≥16 years who participated 1977-79 in a community based health study and were anonymously linked with the Swiss National Cohort until the end of 2008. During follow-up, 727 participants died of CVD. Based on the ESC SCORE methodology (Weibull regression), we used age, sex, blood pressure, smoking, and fasting glucose or total cholesterol. The mean Brier score (BS), area under the receiver-operating characteristic curve (AUC) and integrated discrimination improvement (IDI) were used for model comparison. We validated our models internally using cross-validation and externally using another data set.

**Results:**

In our models, the p-value of total cholesterol was 0.046, that of glucose was p < 0.001. The model with glucose had a slightly better predictive capacity (BS: 2216x10^-5^ vs. 2232x10^-5^; AUC: 0.9181 vs. 0.9169, IDI: 0.009 with p-value 0.026) and could well discriminate the overall risk of persons with high and low concentrations. The external validation confirmed these findings.

**Conclusions:**

Our study suggests that instead of total cholesterol glucose can be used in models predicting overall CVD mortality risk.

## Introduction

Estimation of individual risk of cardiovascular disease (CVD) is traditionally based on age, sex, smoking status, blood pressure and cholesterol parameters. Based on these variables, the SCORE (Systematic COronary Risk Evaluation) risk model predicts fatal CVD events [1]. However, in our general population sample from Switzerland (National Research Project 1A, NRP 1A) with a follow-up time of 32 years [2], total cholesterol performed only poorly in this CVD model. Moreover, lowering cholesterol for primary prevention of CVD has recently been challenged [3]. In contrast, there is increasing evidence for glucose parameters being independent CVD risk factors [4]. This variable was also available in NRP 1A, and we tested the use glucose instead of total cholesterol for risk prediction with the SCORE approach which has a CVD risk chart as central visual element. This chart allows the inclusion of not more than five variables [1]. We validated our results internally and externally [5]. As in other developed countries, in Switzerland, CVD mortality has substantially decreased over the past three decades, resulting in internationally very low figures [6,7]. Despite this, Switzerland has fairly high total cholesterol concentrations compared to other countries [8]. The prevalence of major CVD risk factors remained relatively stable or only slightly declined over the past three decades [6,9].

## Methods

### Study population

Risk factor data stems from men and women aged ≥16 years who participated in the National Research Program 1A (NRP 1A), a community health promotion initiative focused on cardiovascular disease prevention and conducted 1977-1979 in Switzerland [2]. We obtained mortality follow-up by anonymously linking the NRP1A data with the Swiss National Cohort (SNC). The SNC encompasses all residents of Switzerland enumerated in the national 1990 or 2000 censuses as well as data from death and emigration registries [10].

Overall 8,008 out of 8,631 original participants (92.8%) could be linked, loss to follow-up 1980-2000 was 5.8%. Median follow-up was 30.8 years (IQR: 22.4 - 31.1 years) and the 95th centile of follow-up was 31.2 years. In total 1,234 men and 1,193 women died, of whom 471 and 466 from CVD [11]. Since there was no census at the end of the study, loss to follow-up after the 2000 census could not be determined, i.e. all 5,019 individuals linked to the 2000 census but not to a succeeding death or emigration record were assumed to have survived. Between 1990 and 2000, no satisfactory link to a mortality record, emigration record or 2000 census record could be found for 6.9% of the persons registered in the 1990 census [10]. A similar proportion of all registered deaths between the 2000 census and the end of 2008 (6.2%) could not be linked to a 2000 census record [10]. Similar figures can be expected for the period 2000-2010.

### Measurements and outcomes

Blood sampling, glucose and cholesterol measurements were described in detail [2]. Briefly, 5ml of blood were sampled and immediately thereafter centrifugated for 10 minutes at 3000 rpm. The plasma was then sent at 4°C to a laboratory in Bern or Geneva where they were analyzed with a Greiner Electronic Selective Analyzer (GSA) II or a ABA 100 (Abbott laboratories). No other lipid parameters were measured. The results of the internal and external quality control were excellent (day-to-day variability based on the lowest age-specific variance was 1.78% and 2.7%) [2]. Some samples were not taken in fasting state. However, exact times of sampling and of last meal were reported. Persons who had a meal within two hours before sampling were excluded (n = 1901). Smoking status was assessed with a self-administered questionnaire. We defined smoking as smoking > =1 cigarette/d. Non-smokers include former and never smokers. Systolic blood pressure was measured with a sphygmomanometer in a sitting position; we took the mean out of two measurements. Persons with values ≥ 160/95 mmHg were referred to their GP [2]. Fatal CVD events were defined according to ICD (International Classification of Diseases) revisions 8 (ICD-8: 390-458, until 1994) and 10 (ICD-10: I00-I99).

### Statistical analyses

After exclusion of persons with missing covariate information, 2,768 men and 3,327 women remained for analysis, of whom 354 and 373 died of CVD. Risk models were calculated using Weibull proportional hazards regression as described [1]. We calculated the 10-year risk of death from CVD. Note that the complete follow-up time is used for model fitting, thus incorporating all possible information from the data, and the desired prediction time of 10 years is chosen afterwards. This prediction time can be chosen arbitrarily once the model is fitted. In separate sensitivity analyses (Weibull) we also calculated the 20- and 30-year risk (Additional file 1: Figures A3 and A4). We also combined glucose and cholesterol together with the other CVD-risk variables in a single model with two strata for sex (Additional file 1: Table A1) and, in an additional model, included the variable "known diabetes".

In order to compare the predictive abilities of the cholesterol and the glucose model, we calculated the cross-validated (leave-one-out) mean Brier score [12], the area under the receiver-operating characteristic curve (AUC) and the integrated discrimination improvement (IDI). A permutation test [13] was used for the comparison of Brier scores from different models, and a Wald test was applied in the case of the IDI [14]. The Brier score measures the mean squared difference between the risk score and the actual outcome. The lower this deviation, the better the respective risk prediction model. We chose the Brier score because it covers both calibration and sharpness of a prediction model. In contrast, the AUC is mainly a measure for the risk score’s ability to classify correctly. The IDI is a measure of improvement in model performance and represents the difference in discrimination slopes in the two competing models.

We additionally used the US-based NHANES (National Health and Nutrition Examination Survey) III data set to validate our proposed risk score externally. From this data set, only Non-Hispanic white participants who provided a fasting blood sample (4,255 persons, 738 CVD deaths) were used for comparison [15]. The 10-year risk of CVD death was calculated for each individual, using the estimated coefficients from our Weibull models. From these risks, the mean Brier score, AUC and IDI were calculated.

Analyses were performed with STATA 11 (Stata Corp, Texas, USA, 2009) and R (R Foundation for Statistics Computing, version 2.14.1).

## Results

The mean age at baseline was 42.4 years (men) and 44.1 years (women). 50.3% of men and 27.7% of women were current smokers. In men and women, respectively, mean systolic blood pressure was 130.2 mmHg and 125.4 mmHg, mean fasting glucose concentration was 5.4 and 5.2 mmol/L. Mean total cholesterol concentration was 6.0 mmol/L in both sexes. Of the 160 persons who reported having diabetes, 98 were treated only using a diabetes diet, 21 took medication and 16 required injections.

As shown in Table 1 the mean Brier score of the model with glucose (2216x10^-5^) and cholesterol (2232x10^-5^) were similar, and no statistically significant difference could be found (p-value 0.287). Since strong explanatory variables (age, sex, smoking, blood pressure) other than glucose or cholesterol remained the same in the two models, the difference between the Brier scores was expected to be small, stressing the comparability of the results from both risk prediction models. The slightly higher AUC of the model with glucose (0.9181 vs. 0.9169) and the joint model (Additional file 1: Table A1) suggests some advantage for glucose. This finding is confirmed by the statistically significant positive value of the IDI (0.009; p-value: 0.026). In separate analyses including all study participants with cholesterol values (i.e. also persons with non-fasting glucose values, n = 7,991), total cholesterol was statistically not significant (p = 0.089).

**Table 1:** Estimated parameters and coefficients of the two models

	**Model with glucose**		**Glucose vs. cholesterol**	**Model with cholesterol**	
	**Estimate (95% CI)**	**P-value**	**Estimate (P-value)**	**Estimate (95% CI)**	**P-value**
α men	−47.7 (−50.7; -44.4)	<0.001		−47.5 (−50.7; -44.4)	<0.001
p men	9.9 (9.2; 10.6)	<0.001		9.9 (9.2; 10.6)	<0.001
α women	−58.8 (−62.7; -54.9)	<0.001		−58.5 (−62.4; -54.6)	<0.001
p women	12.4 (11.5; 13.2)	<0.001		12.5 (11.7; 13.1)	<0.001
Current smoking	0.37 (0.20; 0.54)	<0.001		0.34 (0.17; 0.51)	<0.001
Systolic blood pressure (mmHg)	0.01 (0.01; 0.02)	<0.001		0.01 (0.01; 0.02)	<0.001
Glucose (mmol/l)	0.10 (0.06; 0.14)	<0.001			
Cholesterol (mmol/l)				0.10 (0.00; 0.20)	0.046
Model comparison					
Brier score (mean, cross-validated)	2216 x 10^-5^		(0.287)	2232 x 10^-5^	
AUC (cross-validated)	0.9181			0.9169	
IDI (glucose vs. cholesterol)			0.009 (0.026)		
Model comparison, external validation*					
Brier score (mean)	7662 x 10^-5^		(0.245)	7744 x 10^-5^	
AUC	0.8766			0.8737	
IDI (glucose vs. cholesterol)			0.029 (<0.001)		

*with data from the third National Health and Nutrition Examination Survey (NHANES III).

*AUC* area under the receiver-operating characteristic curve; *IDI* integrated discrimination improvement.

**Figure 1:**
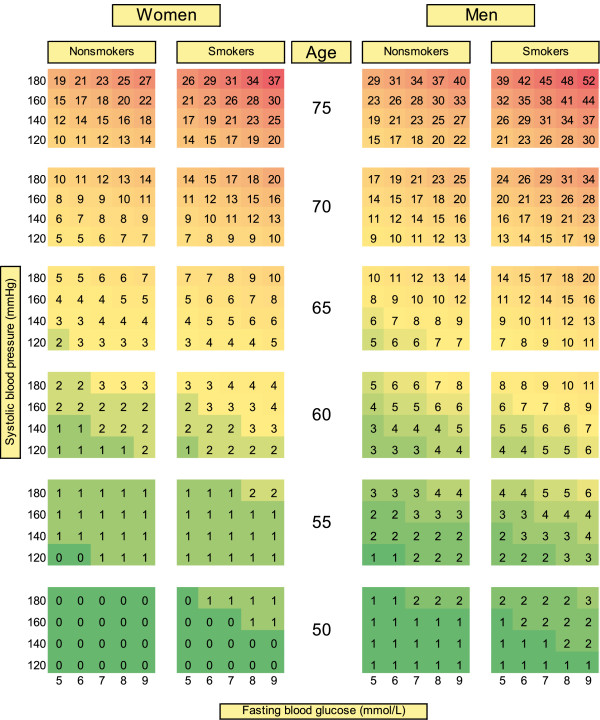
**Chart for absolute 10-year risk of fatal cardiovascular disease based on the model using blood glucose instead of cholesterol, 6,095 participants of the NRP1A study conducted in Switzerland in 1977-79, ages >16 years at baseline.** NRP1A: National Research Program 1A, entire population with full follow-up is considered. Each risk percentage is calculated using a combination of given risk factor values. E.g., a man aged 65, smoker, with a systolic blood pressure of 180 and a fasting blood glucose of 6 mmol/L has an absolute risk of fatal CVD of 15%.

As shown in Figure 1, glucose can well discriminate persons at high and low CVD risk at virtually all ages and blood pressure levels, irrespective of smoking status. Particularly at older ages, there were strong synergistic effects of the combination of glucose with smoking and in particular with blood pressure. Sensitivity analyses showed that glucose remained significant (p < 0.001) in a joint model with cholesterol while cholesterol was not significant (p = 0.073). Thus, cholesterol could not contribute to the explanation of the association between risk factors and mortality when glucose was included in the same model (Additional file 1: Table A1). Stratifying risk estimation based on blood glucose for persons with high and low cholesterol concentration did also not result in a substantial improvement in risk estimation (Additional file 1: Figure A1). In the common model with cholesterol, further adjustment for self-reported diabetes ("known diabetes") had no significant impact on estimates (not shown).

The external validation using the NHANES III data, confirmed the overall pattern (Table 1): The results with glucose and cholesterol are comparable and also suggest slightly better predictive performance of the model with glucose (Brier score: 7662x10^-5^ vs. 7744x10^-5^ with p-value 0.245; AUC: 0.8766 vs. 0.8737; IDI:0.029 with p-value < 0.001).

## Discussion

Our models based on data from Switzerland suggest that instead of cholesterol, blood glucose could be used for prediction of fatal CVD. In fact, glucose performed similarly in our CVD risk prediction model. In contrast to cholesterol, the coefficient of glucose remained statistically significant in a joint model including both glucose and cholesterol. Traditional CVD risk models do not consider glucose. The PROCAM and the Framingham models include information about diabetes (yes/no) but this does not sufficiently map the potential impact of blood glucose on CVD [16,17]. As shown by us and others, there is a mortality gradient below the threshold for diabetes. Mortality risk increases at concentrations around ≥6 mmol/L [4,18,19]. This threshold may be even lower when other CVD risk factors, e.g. high blood pressure or smoking are present [20,21]. Such combinations are presented in our chart. The advantage of using blood glucose in a continuous instead of a dichotomized form is that the chart could be used by physician to prevent or delay the onset of diabetes in persons with prediabetic glucose concentrations and reduce morbidity and premature death. It could be used to motivate individuals to follow lifestyle recommendations and to increase compliance. In fact, glucose parameters can be improved with physical activity, weight management and healthy diet and thus open doors for lifestyle recommendations and underline their significance [22-24]. Prediabetes can also be effectively treated with Metformin which can decrease the rate of conversion from prediabetes to diabetes [22,25,26]. Approaching persons early in the pathway to diabetes may be much more effective than treating them when diabetes is established. This is not possible when the risk associated with increased glucose concentrations is only considered dichotomously (no diabetes / diabetes).

Using glucose may also have practical advantages, because it can easily and reliably be measured e.g., from capillary blood or even from saliva [27]. The assessment of total and particularly HDL cholesterol is more extensive and in case of cholesterol ratio requires two measurements. Moreover, blood glucose is routinely assessed in clinical practice or self-controlled by many patients. This enables a straightforward and continuous monitoring of CVD risk.

The impact of cholesterol as CVD risk factor in persons with low CVD risk has been questioned recently [3]. In our study, lag of time between measurement of risk factors and death was up to 32 years. Since only baseline information was available, we cannot determine whether participants were treated with lipid lowering medication or diabetes medication. In Switzerland, cholesterol-lowering statin drugs are used since the end of the 1980s. In 2002 and 2007 respectively, 5.0% and 7.4% of men and 3.4% and 4.9% of women reported daily treatment of increased cholesterol levels [28]. Anti diabetic drugs are used since 1960 in Switzerland (Metformin). In 2007, 2.9% of men and 2.0% of women reported being treated daily for diabetes [28].

Our study has several limitations. CVD risk factors could only be assessed once (at baseline) and there is no information about changes in exposures. The found association with mortality can thus be assumed to be conservative, i.e. an underestimation of the “real” magnitude of the effect. Previous sensitivity analyses showed, however, that point estimates for relative risks of CVD risk factors only moderately differed if 10 or 15 years were considered instead of the full follow-up time [29]. An underestimation can also be expected from the fact that we may have included non-fasting persons. In fact, restricting to persons who did not eat four hours (instead of two) before blood sampling resulted in higher risks in virtually all strata (see Additional file 1: Figure A2).

Another limitation is the fact that the competing risk of non-CVD mortality was not accounted for in our models. Estimated baseline hazards are necessary for our risk calculations and can be obtained by using Weibull regression (as performed). Unfortunately, this estimation is not possible with standard competing risks methodology. Some bias might be introduced by not considering competing risks, however, this bias is generally supposed to be small.

A part of the original study participants could not be linked (7.2%), emigrated (2.7%) or was lost to follow-up (3.8%, not considering the unknown figure for 2000-08). However, even on the long run, this proportion remains rather modest. In line with other health surveys, the NRP 1A participants were most likely healthier than the general population [30]. Our end-point was limited in the sense that we were restricted to fatal CVD. CVD mortality data from death certificates is reliable for young and middle aged persons but may cause misclassification in elderly [31]. However, overall, in Switzerland, the validity (58-77%) and reliability (90-97%) of coding of CVD deaths from death registry may be regarded as acceptable [32]. The appearance of the traditional ESC chart is predefined and we wish to adhere to it. Therefore we could not display glucose AND cholesterol in the same chart [33]. In our population, we found no evidence for a substantial advantage of including cholesterol in addition to glucose (see Additional file 1: Table A1, Figure A1).

## Conclusion

Our results are a hint that established CVD risk prediction models are worth being revisited. However, the performance of glucose vs. cholesterol needs to be validated with populations from countries outside Europe and the USA.

## Abbreviations

CVD: Cardiovascular disease; ICD: International classification of diseases; NRP1A: National research program 1A; PROCAM: Prospective cardiovascular münster study; SCORE: Systematic COronary risk evaluation; SNC: Swiss national cohort.

## Competing interests

The authors have no competing interests to declare.

## Authors’ contribution

DF conceived the study, designed tables and figures and mainly wrote the manuscript. JB assisted in the record linkage, conducted all statistical analyses and wrote a part of the manuscript. MB added background knowledge and improved the manuscript by repeated readings and rephrasing as well as critical discussions of the intellectual content. All authors read and approved the final manuscript.

## Supplementary Material

Additional file 1: Table A1 Hazard ratios of considered risk factors based on a model* including glucose AND cholesterol; **Figure A1**. Chart for absolute 10-year risk of fatal cardiovascular disease based on the model using blood glucose (five values) and cholesterol (dichotomized), 6,095 participants of the NRP1A study conducted in Switzerland in 1977-79, ages >16 years at baseline; **Figure A2**. Chart for absolute 10-year risk of fatal cardiovascular disease based on the model using blood glucose instead of cholesterol, 3,217 participants of the NRP1A study with fasting time > 4 hours, Switzerland, 1977-79 (baseline), ages >16 years at baseline; **Figure A3**. Chart for absolute 20-year risk of fatal cardiovascular disease based on the model using blood glucose instead of cholesterol, 6,095 participants of the NRP1A study conducted in Switzerland in 1977-79, ages >16 years at baseline; **Figure A4**. Chart for absolute 30-year risk of fatal cardiovascular disease based on the model using blood glucose instead of cholesterol, 6,095 participants of the NRP1A study conducted in Switzerland in 1977-79, ages >16 years at baseline. Click here for file
