# Supplementary material for: Blood glucose may be an alternative to cholesterol in CVD risk prediction charts
Source: Cardiovasc Diabetol. 2013 Jan 25;12:24. doi: 10.1186/1475-2840-12-24 (PMC3599220; doi:10.1186/1475-2840-12-24)
Supplement: Additional file 1: Table A1 — Hazard ratios of considered risk factors based on a model* including glucose AND cholesterol; Figure A1. Chart for absolute 10-year risk of fatal cardiovascular disease based on the model using blood glucose (five values) and cholesterol (dichotomized), 6,095 participants of the NRP1A study conducted in Switzerland in 1977-79, ages >16 years at baseline; Figure A2. Chart for absolute 10-year risk of fatal cardiovascular disease based on the model using blood glucose instead of cholesterol, 3,217 participants of the NRP1A study with fasting time > 4 hours, Switzerland, 1977-79 (baseline), ages >16 years at baseline; Figure A3. Chart for absolute 20-year risk of fatal cardiovascular disease based on the model using blood glucose instead of cholesterol, 6,095 participants of the NRP1A study conducted in Switzerland in 1977-79, ages >16 years at baseline; Figure A4. Chart for absolute 30-year risk of fatal cardiovascular disease based on the model using blood glucose instead of cholesterol, 6,095 participants of the NRP1A study conducted in Switzerland in 1977-79, ages >16 years at baseline. [file 1475-2840-12-24-S1.docx]

**Additional file**

**Table A1.** Hazard ratios of considered risk factors based on a model* including glucose AND cholesterol

|  |  |  |  |  |  |  |  |  |  |  |  |  |  |  |  |
| --- | --- | --- | --- | --- | --- | --- | --- | --- | --- | --- | --- | --- | --- | --- | --- |
|  | Model 1 | | |  | Model 2 | | |  | Model 3 | | |  | Model 4 | | |
|  | HR | p-value | z-value |  | HR | p-value | z-value |  | HR | p-value | z-value |  | HR | p-value | z-value |
| Current smoking (yes/no) | 1.42 | <0.001 | 4.13 |  | 1.45 | <0.001 | 4.31 |  | 1.41 | <0.001 | 3.98 |  | 1.44 | <0.001 | 4.18 |
| Systolic blood pressure (mmHg) | 1.01 | <0.001 | 6.51 |  | 1.01 | <0.001 | 6.14 |  | 1.01 | <0.001 | 6.32 |  | 1.01 | <0.001 | 8.15 |
| Glucose (mmol/l) |  |  |  |  | 1.11 | <0.001 | 4.96 |  |  |  |  |  | 1.11 | <0.001 | 4.84 |
| Cholesterol (mmol/l) |  |  |  |  |  |  |  |  | 1.11 | 0.046 | 2 |  | 1.10 | 0.073 | 1.79 |
|  |  |  |  |  |  |  |  |  |  |  |  |  |  |  |  |

*Weibull regression model with two strata for sex

**Figure A1**. Chart for absolute 10-year risk of fatal cardiovascular disease based on the model using blood glucose (five values) and cholesterol (dichotomized), 6,095 participants of the NRP1A study conducted in Switzerland in 1977-79, ages >16 years at baseline

**Figure A2**. Chart for absolute 10-year risk of fatal cardiovascular disease based on the model using blood glucose instead of cholesterol, 3,217 participants of the NRP1A study with fasting time > 4 hours, Switzerland, 1977-79 (baseline), ages >16 years at baseline

NRP1A: National Research Program 1A, entire population with full follow-up is considered

**Figure A3**. Chart for absolute 20-year risk of fatal cardiovascular disease based on the model using blood glucose instead of cholesterol, 6,095 participants of the NRP1A study conducted in Switzerland in 1977-79, ages >16 years at baseline

NRP1A: National Research Program 1A, entire population with full follow-up is considered

**Figure A4**. Chart for absolute 30-year risk of fatal cardiovascular disease based on the model using blood glucose instead of cholesterol, 6,095 participants of the NRP1A study conducted in Switzerland in 1977-79, ages >16 years at baseline

NRP1A: National Research Program 1A, entire population with full follow-up is considered
